# Identification of Common Genetic Variation That Modulates Alternative Splicing

**DOI:** 10.1371/journal.pgen.0030099

**Published:** 2007-06-15

**Authors:** Jeremy Hull, Susana Campino, Kate Rowlands, Man-Suen Chan, Richard R Copley, Martin S Taylor, Kirk Rockett, Gareth Elvidge, Brendan Keating, Julian Knight, Dominic Kwiatkowski

**Affiliations:** 1 University Department of Paediatrics, John Radcliffe Hospital, Oxford, United Kingdom; 2 Wellcome Trust Centre for Human Genetics, University of Oxford, Oxford, United Kingdom; 3 Wellcome Trust Sanger Institute, Hinxton, United Kingdom; The Wellcome Trust Sanger Institute, United Kingdom

## Abstract

Alternative splicing of genes is an efficient means of generating variation in protein function. Several disease states have been associated with rare genetic variants that affect splicing patterns. Conversely, splicing efficiency of some genes is known to vary between individuals without apparent ill effects. What is not clear is whether commonly observed phenotypic variation in splicing patterns, and hence potential variation in protein function, is to a significant extent determined by naturally occurring DNA sequence variation and in particular by single nucleotide polymorphisms (SNPs). In this study, we surveyed the splicing patterns of 250 exons in 22 individuals who had been previously genotyped by the International HapMap Project. We identified 70 simple cassette exon alternative splicing events in our experimental system; for six of these, we detected consistent differences in splicing pattern between individuals, with a highly significant association between splice phenotype and neighbouring SNPs. Remarkably, for five out of six of these events, the strongest correlation was found with the SNP closest to the intron–exon boundary, although the distance between these SNPs and the intron–exon boundary ranged from 2 bp to greater than 1,000 bp. Two of these SNPs were further investigated using a minigene splicing system, and in each case the SNPs were found to exert *cis*-acting effects on exon splicing efficiency in vitro. The functional consequences of these SNPs could not be predicted using bioinformatic algorithms. Our findings suggest that phenotypic variation in splicing patterns is determined by the presence of SNPs within flanking introns or exons. Effects on splicing may represent an important mechanism by which SNPs influence gene function.

## Author Summary

Genetic variation, through its effects on gene expression, influences many aspects of the human phenotype. Understanding the impact of genetic variation on human disease risk has become a major goal for biomedical research and has the potential of revealing both novel disease mechanisms and novel functional elements controlling gene expression. Recent large-scale studies have suggested that a relatively high proportion of human genes show allele-specific variation in expression. Effects of common DNA polymorphisms on mRNA splicing are less well studied. Variation in splicing patterns is known to be tissue specific, and for a small number of genes has been shown to vary among individuals. What is not known is whether allele-specific splicing events are an important mechanism by which common genetic variation affects gene expression. In this study we show that allele-specific alternative splicing was observed in six out of 70 exon-skipping events. Sequence analysis of the relevant splice sites and of the regions surrounding single nucleotide polymorphisms correlated with the splicing events failed to identify any predictive bioinformatic signals. A genome-wide study of allele-specific splicing, using an experimental rather than a bioinformatic approach, is now required.

## Introduction

The sequencing of the human genome [1,2] and subsequent work describing sequence variation amongst human populations [3] has provided the necessary resources for large-scale studies of the effects of genetic variation on human gene expression. Identifying functionally important variation has the potential for increasing understanding of gene regulation and for providing efficient markers to study the effects of variation in gene expression on human disease risk [4]. Experimentally demonstrating the potential functional effects of DNA polymorphism is difficult, as these effects may be both tissue and stimulus specific. Significant efforts have focused on transcriptional regulation, because of the strong suspicion that the majority of human phenotypic variation is due to regulatory variants [5,6]. Novel allele-specific transcript quantification approaches to candidate genes [7,8] have been employed, along with broader approaches to investigate the absolute levels of expression of thousands of genes [9,10]. Using these methods, several *cis*-acting SNPs that correlate with gene expression have been identified. However, fine mapping these effects and determining the mechanisms underlying the associations has been more difficult [11].

In this study, we used a different approach—that of evaluating effects on splicing efficiency—to study the effects of common genetic polymorphism on gene function. The vast majority of human genes are comprised of three or more exons that need to be efficiently spliced together to form mature mRNA. Variation in this process occurs naturally and is thought to be an important mechanism whereby different protein products can be derived from the same gene sequence [12]. Single base changes that affect splicing can have dramatic effects on gene function and can cause disease, usually because the splice mutation results in a shift in the amino acid reading frame. Most commonly observed alternative splicing events preserve the reading frame and have more subtle effects on protein function [13]. There are an increasing number of examples in which the genetically determined modulation of alternative splicing has been implicated in common complex disease traits, such as the associations between the G protein-coupled receptor *(GPRA)* and asthma susceptibility [14]*,* cytotoxic T lymphocyte antigen 4 *(CTLA4)* and autoimmune disease [15], and the CD45 (leucocyte common) antigen and infectious and autoimmune diseases [16,17]. The potential effects of common SNPs on splicing isoforms have been suggested by bioinformatic analysis of expressed sequence tags [18]. In a small number of genes, these potential effects have been demonstrated experimentally [19–21]. Here, we used lymphoblastoid cell lines (LCLs) from the Centre d'Etude du Polymorphisme Humain (CEPH) as an experimental model system to investigate the relationship between variation in simple cassette exon splicing events and genotypic diversity. We sought to determine (1) whether individual variation in splicing patterns was commonly observed, (2) if any observed phenotypic variation could be explained by genetic differences among individuals, and (3) whether any genetic differences could be localised and the functional element identified.

## Results

### Inter-individual Variation in Splice Pattern

Our initial aim was to investigate whether there was variation among individual LCLs in simple cassette exon events. These events were defined as the occurrence of complete exon skipping in two or more mRNA isoforms. We used a strategy of exon selection that we believe increased the likelihood of detecting allele-specific effects on alternative splicing. We argue that for genes in which common SNPs affect splicing, at least two mRNA transcript isoforms of that gene will be relatively commonly observed. Conversely, where only one transcript isoform has been observed and documented, the likelihood of a SNP-related splicing event is reduced. We identified 2,281 simple cassette exon events from the European Bioinformatics Institute Alternative Splicing Database (EBI-ASD) in which each transcript isoform had been observed in at least two clone libraries. From these, we selected the 250 genes with the highest expression levels in LCLs as detected by global microarray analysis. We carried out reverse transcriptase PCR (RT-PCR) analysis of these 250 genes and found that in LCLs both transcript isoforms were present in 70 (28%) of the genes.

We proceeded to investigate whether the amount of different isoforms varied between 22 different LCLs. Of the 70 events that produced both full-length and exon-skipped products, we found that 18 (26%) showed significant variation among cell lines, in which at least one cell line showed a ratio of PCR products that differed by more than 10% of the mean value for the entire sample set of 22 cell lines (10% difference in relative abundance is the lower limit of sensitivity of the detection assay). These 18 events were retested using RNA derived from an independent round of cell culture. Six events, centered around genes *CASP3, CD46, IFI16, RBM23, SH3YL1,* and *ZDHHC6,* demonstrated repeatable and consistent variation of the splicing pattern among different cell lines. The genes and exons for each of these six events are listed in Table 1. None of the skipped exons resulted in a shift in the reading frame of the mRNA. We did not investigate the remaining 12 events; these provided inconsistent results, as splicing isoforms were present only at very low intensity or in only one or two cell lines.

**Table 1:**
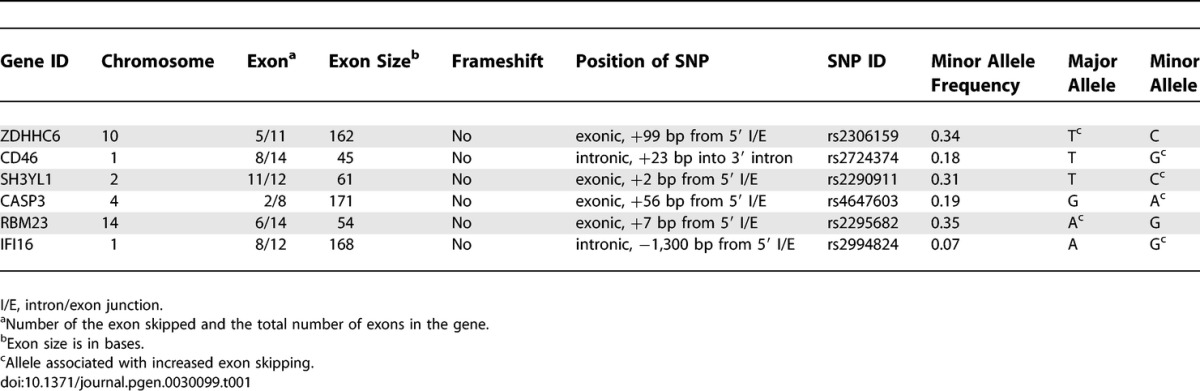
Details of the Alternatively Spliced Exons and Associated SNPs

### SNP Genotype Predicts Splice Pattern

**Figure 1:**
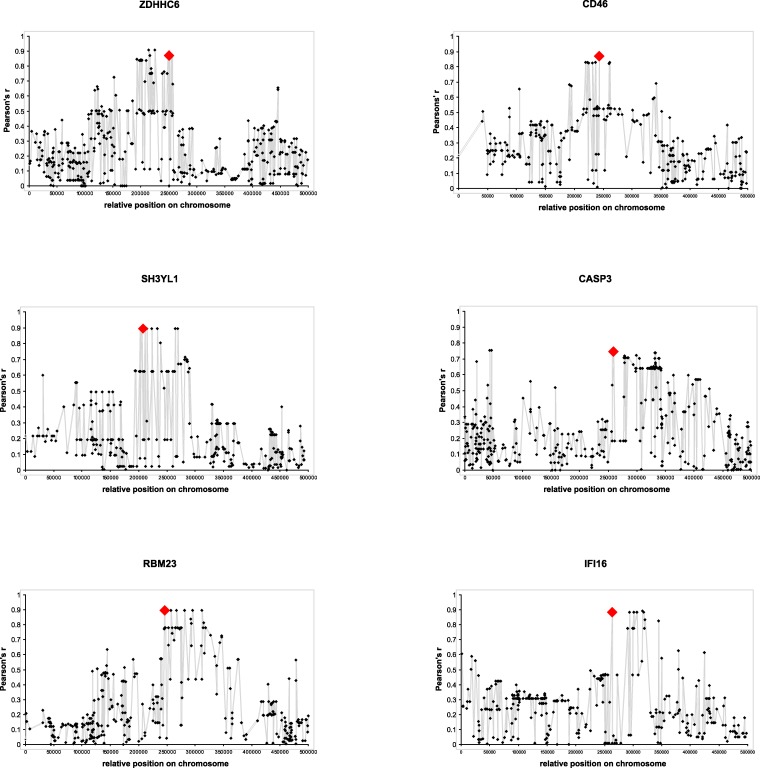
Correlation of Splice Pattern with SNP Genotype In each graph, Pearson's *r* is plotted against relative chromosomal SNP position for all SNPs identified by the HAPMAP consortium within a 500-kb region surrounding the relevant gene. For each graph, the HUGO gene name is given and the SNP nearest to the intron–exon boundary at either the 5′ or 3′ end of the skipped exon is highlighted. For each gene this SNP was either within the skipped exon or in the flanking intron (see Table 1). Each of the six genes is between 15 and 50 kb in size.

We next investigated the relationship between DNA sequence variation and observed differences in splice isoforms among LCLs. We looked at the correlation between SNP genotype and splicing pattern over the 500-kb region surrounding each of the six splicing events that showed consistent variation among the LCLs. Two sources of SNP genotyping data were used. First, we analysed SNP genotypes from the International HapMap Project [3]. Second, we resequenced the skipped exons and 150 bp of the flanking introns for each event in each of the 22 cell lines. Resequencing did not identify any SNPs that were not already identified on the HapMap resource. Approximately 350 SNPs were available for each gene at an average density of 0.7 SNPs per kb. For each of the six events, highly significant correlations between SNPs and the observed splicing pattern were identified (Figure 1). The maximum values for the Pearson's statistic were 0.76 (*p* < 10^−4^) for the *CASP3* event and over 0.86 (*p* < 10^−6^) for the other five events. For five of the six events, the SNP nearest the intron–exon boundary showed the strongest correlation with splicing pattern. For the *ZDHHC6* event, a slightly higher value for the Pearson's statistic was seen for a group of three SNPs lying over 50 kb away from the gene and in very strong linkage disequilibrium (LD) (R^2^ = 0.95) with the SNP nearest the intron–exon boundary. When we studied the *ZDHHC6* SNP nearest the intron–exon boundary in the minigene system (see below), we observed a direct effect of this SNP on splicing efficiency, suggesting that this SNP, rather than the more distant group of three SNPs, was responsible for the observed variation in splice pattern. To test whether the identified SNP accurately predicted the variation in splice pattern, we selected a new set of nine unrelated LCLs in which there was at least one example of each of the possible SNP genotypes. For each of the six genes, the splicing pattern observed was accurately predicted by the SNP genotype (Figure S1).

**Figure 2:**
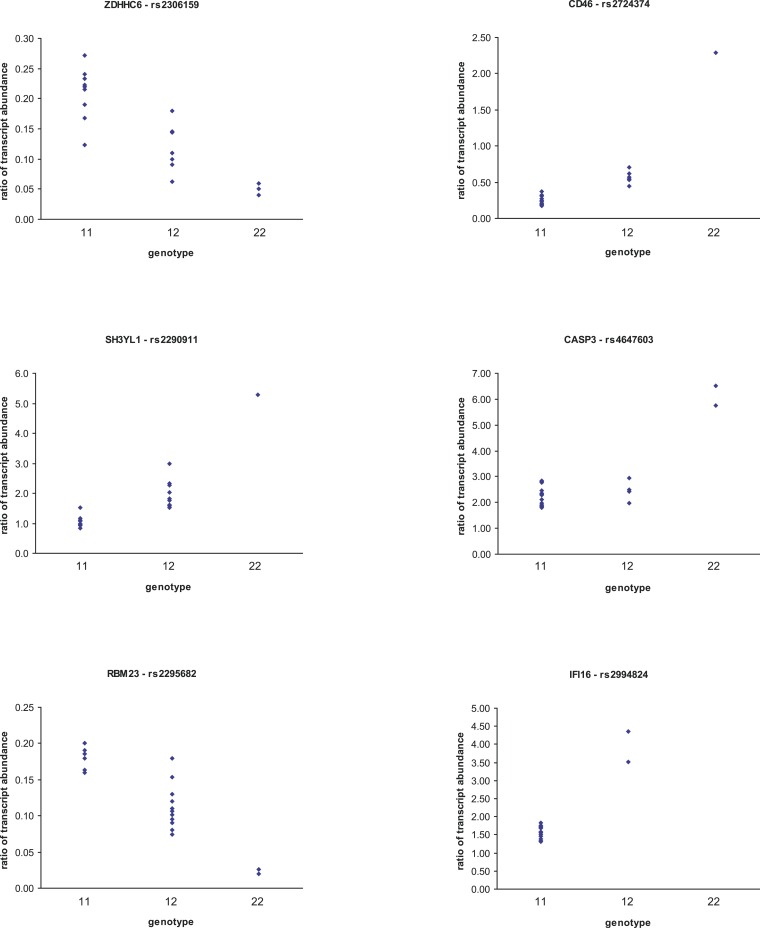
Relative Transcript Abundance Grouped by SNP Genotype The ratio of transcript abundance (skipped product/full-length product) for each of the six alternative splicing events that showed consistent variation between different individuals is shown. For each gene, the ratios are grouped by the genotype of the SNP nearest the intron–exon junction of the splicing event.

The correlations between splice pattern and individual SNPs are highly significant even after allowing for correction for multiple comparisons. If we use a simple Bonferroni correction for the 350 SNPs that were tested for each simple cassette exon event, all results remain significant at the 0.05 level. This level of correction is overly conservative, since the LD relationship among the SNPs means that they are not independent of one another. Furthermore, it is remarkable that for five of the six events it is the SNP closest to the intron–exon boundary that is the strongest predictor of splicing phenotype. When we analysed the effects of the SNP nearest the intron–exon boundary of each event, a clear effect of genotype on relative abundance of each product was found. The measured ratios of the two splice products are plotted by genotype in Figure 2. The magnitude of allele-specific effect was similar for each gene and represented an approximately a 2-fold additive effect on the ratio of splice isoforms. In four out of the six events, the minor allele was associated with an increased abundance of mRNA with the exon-skipping event. For the other two (*RMB23* and *ZDHHC6*), the minor allele was associated with an increased abundance of the full length mRNA. These data suggest that *cis*-acting variation is directly modulating the pattern of observed alternative splicing at these loci.

For five of the six events, there is an apparent dose-dependent effect with larger effects seen in homozygotes compared with heterozygotes. For the *CASP3* event, the effect of the SNP on relative abundance of the two transcript isoforms was only seen in the homozygous state. The effect of the *CASP3* SNP in the homozygous state was nevertheless clear-cut and repeatable. We were puzzled as to why we were unable to detect an effect when the SNP was present on only one chromosome since a *cis*-acting mechanism of action seems most likely. One possible explanation is that when only one chromosome carries the splicing SNP, up-regulation of expression from the other chromosome compensates for the loss of the full-length product. To test this hypothesis, we quantified the relative abundance of *CASP3* transcripts derived from each chromosome in LCLs from 16 unrelated CEPH individuals heterozygous for the rs4647603 *CASP3* exonic SNP. These experiments showed that all 16 heterozygous individuals had a higher relative abundance of transcripts containing the rs4647603 G allele compared to those with the A allele (on average 2.7 times more G than A, Figure S2). In the homozygous state, the A allele is associated with increased exon skipping. In the heterozygous state, the relative proportions of the full-length and exon-skipped products appear unchanged. The higher relative abundance of *CASP3* transcripts containing the rs4647603 G allele suggests increased expression of *CASP3* derived from this chromosome and is consistent with an effect of the rs4647603 A allele on splicing in the heterozygote state.

### Splice Site Analysis

**Figure 3:**
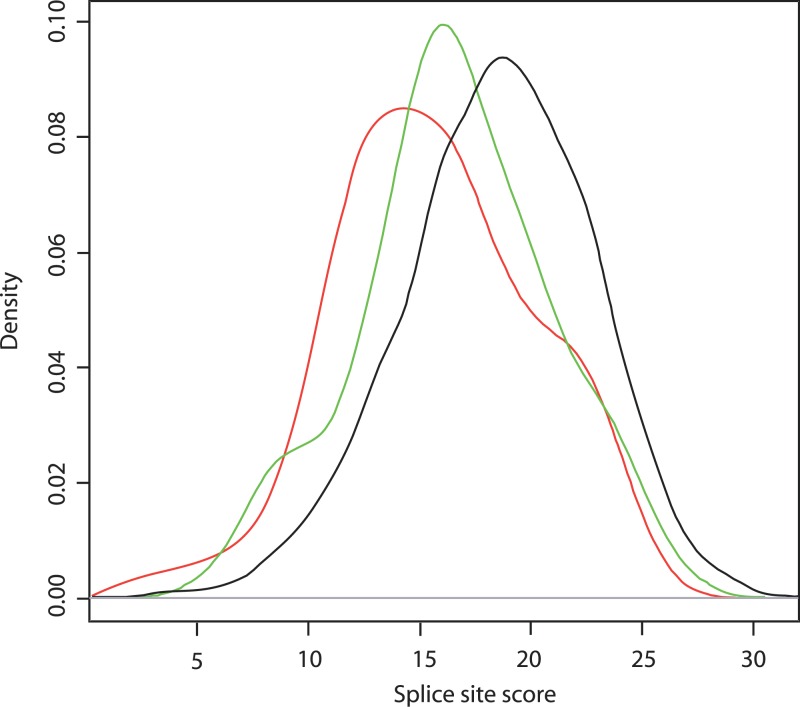
Comparison of Splice Site Scores Using a Density Plot The distribution of splice site scores was compared using a density plot generated using the statistical package R (http://cran.uk.r-project.org). Each line shows the distribution of splice site scores for three different sets of exons: the black line (set A) shows scores for a genome-wide set of constitutive exons (n=7431), the green line (set B) shows the scores from the experimental test set of exons predicted to be skipped but that did not show skipping in the CEPH system (*n* = 180), and the red line (set C) represents the scores from the experimental test set that were predicted to be skipped and that actually demonstrated skipping in the CEPH system (*n* = 70). The distributions of splice site scores for set A (mean 18.5) differed from set B (mean 16.6) and set C (mean score 15.7), *p* < 0.0001 for both comparisons.

Splice site signal scores from the donor and acceptor sites of the test exons predicted to show alternative splicing were compared with those from a genome-wide set of constitutively spliced exons (Figure 3). As a group, the test exons had significantly (*p* = 1 × 10^−5^) weaker splice site signal scores than those from constitutive exons. The difference was greatest for the exons in which alternative splicing was experimentally demonstrated. The effect was seen in both donor and acceptor sites and was slightly more pronounced at the donor sites. Although the differences in splice site strength were statistically significant, there was extensive overlap in splice site scores between the groups (Figure 3).

The potential effects on exonic splice enhancer strength of the four exonic SNPs shown to correlate with splice pattern were tested using four different prediction algorithms (see Materials and Methods). For two SNPs, no effects were predicted by any of the four models tested. For the other two SNPs, the results were contradictory (different models showed both increased and decreased splice enhancer activity).

There were no differences in the number of SNPs in the 50-bp regions around the intron–exon junction for the 180 exons that did not show alternative splicing in our experimental model, compared to the 70 exons that did. This suggests that using the position of known SNPs to select for exons with allele-specific splicing patterns is unlikely to be fruitful. Furthermore, of the six exons that showed allele-specific splicing patterns, three showed splicing patterns that were correlated with SNPs situated more than 50 bases from the intron–exon junctions.

### Minigene Analysis Confirms Modulation of Splicing by SNP Genotype

**Figure 4:**
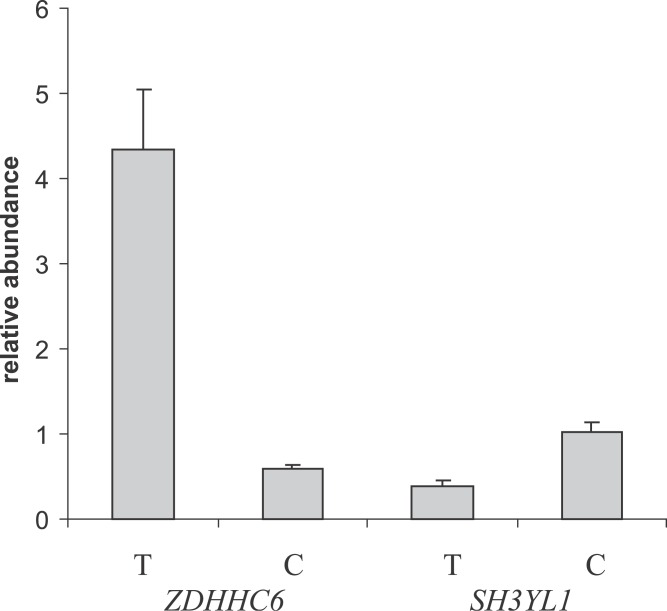
Minigene Transcript Analysis The graph shows the relative abundance of the two transcripts derived from the minigene plasmid, expressed as a ratio of the shorter transcript (with the test exon skipped) to the longer “full length” transcript. Data shown are means of four measurements with confidence intervals. For each test exon there are significant differences in exon exclusion between the two tested allelic variants (T or C for each gene).

To investigate whether SNP genotype directly defined splice isoform pattern, we carried out minigene analysis in two genes. In *ZDHHC6,* the test SNP was situated in the middle of the exon, 99 bp away from the intron–exon boundary. In *SH3YL1,* the test SNP was also exonic, but in this case only 2 bp away from the intron–exon boundary. For each gene, we independently cloned two fragments that differed only by the alleles of the SNP correlated with exon skipping. Each fragment consisted of the alternatively spliced exon plus 180 bp of intronic sequence on each side. The fragments were inserted into a minigene splicing vector that was used to transfect HEK293T cells. After 48 h, mRNA was extracted from the cells and the relative abundance of mRNA (full length and alternative spliced) transcripts derived from the minigene plasmid was determined. For both genes we observed that the SNP allele associated with increased exon skipping in the LCL experiments was also associated with increased exon skipping in the minigene system (Figure 4). For four genes (*SH3YL1, ZDHHC6, IFI16,* and *RNPC4*) there were between four and ten SNPs identified on the HAPMAP resource that were in complete LD with the SNP nearest the intron–exon boundary. All but one of these SNPs lay more than 2 kb away from the exon of interest and are not testable using the minigene system. We cannot discount an effect of these more distant SNPs on the observed allele-specific splicing event.

## Discussion

This study describes reproducible phenotypic variation in splicing among individuals, in each case arising from a simple cassette exon event that is associated with genotypic variation in SNPs close to the corresponding intron–exon boundaries. Our starting point was to screen for phenotypic variation in splicing in 22 lymphoblastoid cell lines, and then to identify SNPs associated with this phenotypic variation. Interestingly, the splicing-associated SNPs identified experimentally in this study did not show any clear difference in position or sequence context from other SNPs that were not associated with splicing variation.

**Figure 5:**
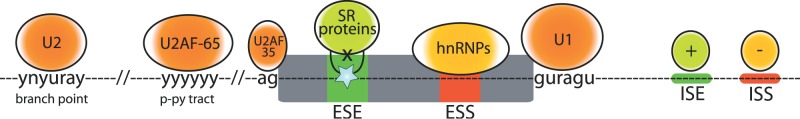
Representation of Classic and Auxiliary Splice Sites and Binding Factors In this example, a SNP (represented by a star) in an exonic splice enhancer sequence has disrupted binding of the SR proteins, reducing the efficiency of exon definition and potentially leading to an alternative splice site being used. Similar disruption could affect exonic splice suppressor, intronic splice enhancer, and intronic splice suppressor elements. ESE, exonic splice enhancer; ESS, exonic splice suppressor; ISE, intronic splice enhancer; ISS, exonic splice suppressor; and p-py, polypyrimidine.

The mechanisms by which alternative splicing is regulated are poorly understood. Exon recognition and splicing requires the presence of basic “classic” splice sites (the branch point, polypyrimidine tract, and the 3′ and 5′ splice sites). The efficiency of the splicing process can be affected in some exons by the presence of auxiliary or modulating elements (Figure 5). The consensus sequences for the known modulating elements are degenerate and frequently found throughout the genome. DNA sequence variation can modulate alternative splicing, and to date attention has focused on disease-causing *cis*-acting mutations affecting the use of constitutive and alternative splice sites, together with *trans*-acting variants that affect the basal splicing machinery and factors regulating splicing [22]. In contrast to mechanistic studies of disease process, our study started from the premise of defining simple cassette exon events in which there was significant variation among a panel of LCLs and relating this to genotypic diversity. We found consistent variation in six out of 70 simple cassette exon events, and for each of these six events we found a clear relationship between genotype and splice phenotype. Analysis of SNPs typed by the International HapMap project and those derived experimentally by resequencing showed that the SNPs with the strongest correlation were those closest to the intron–exon boundaries of the splicing events. For two of the SNPs we carried out minigene experiments, and both showed *cis-*acting effects on gene splicing using this system.

It is perhaps not surprising that we were unable to detect any specific patterns in the sequence context of the six SNPs identified in this study, given the apparent degenerate nature of consensus sequences that bind splice modulator proteins. Overall the splice-site strengths of the exons that were predicted to be skipped by the EBI-ASD database were weaker than those of constitutive exons, and those that we were able to demonstrate to have alternative splicing in our experimental system had the weakest splice site strength. However, there was significant overlap among the groups, and splice site strength cannot be used to identify the most likely exons to study. Equally, the presence of SNPs close to the intron–exon boundaries did not differ between those exons that did and did not show alternative splicing, suggesting that selecting exons to study according to whether there is a “splice site SNP” (defined for example on Ensembl as a SNP lying within 10 bp of the intron–exon junction) will not enrich for those SNPs that actually affect the splicing process. Only two out of the six SNPs identified in this study were within 10 bp of an intron–exon junction. The exonic SNPs that correlated with splice pattern in this study showed no consistent effects on splice enhancer strength using four different predictive models. Thus, the sequence context or position of the SNPs would not identify those likely to influence splicing efficiency. A different approach to identify allele-specific alternative splicing events that does not rely on the sequence context or the position of SNPs is to identify allele-specific RNA isoforms from EST databases [18]. This approach requires the presence of an exonic SNP not involved in the alternative splicing event to be in high LD with the functional splicing SNP and limits its broad applicability. When applied to our data using HAPMAP SNPs, only the events in *ZDHHC6* and *RBM23* have the potential of being identified. The EST method is prone to false-positive results, particularly for low frequency SNPs, if there are insufficient representative ESTs available in the database. We suggest that an experimental approach, rather than a bioinformatic approach, will be necessary to identify splicing phenotype-associated SNPs, at least until more is learned about how these SNPs exert their functional effects. We believe that identifying alternative splicing events is the essential first step in this experimental approach. While splice enhancers and suppressors are found in constitutive exons and their flanking introns, these exons by definition are not observed to show alternative splicing. This suggests either that no SNPs occur within functionally important splice elements or that the splice enhancer/suppressor signals are not required for the accurate splicing of these exons. The relative positions and sequence context of experimentally identified splicing SNPs can be used to refine predictive algorithms and may provide new insights into which of the many exonic and intronic splice modulator sequences present in every gene are functionally important in regulating the splicing process.

Our method of isoform quantification and pooling strategy meant that our ability to detect rare events was limited. Dilution experiments determined that both the full-length and exon-skipped transcript products were detectable even when their starting concentrations differed by 100-fold. Thus, provided that both transcripts were present in at least one of the 22 cell lines, and the minor transcript was present at an abundance of 30% or greater, the event would be detected. If the rare transcript was present in three or more cell lines, the sensitivity increased to a lower abundance of 10%. The method we used is not readily scalable to whole genome analysis. Microarray-based approaches to the analysis of alternative splicing have been published [23,24]. These approaches can analyse the splicing patterns of many thousands of exons and have been used to distinguish splicing patterns seen in different tissues. Interpretation is complex, and for some arrays sensitivity is low and false positive rates are high. Although it is likely that the technology will improve, these approaches have not yet been shown to have the sensitivity to detect the level of variation we observed in this study, particularly for low-abundance isoforms. The advantage of the system we describe is targeted amplification of the splicing event of interest, which we believe provides greater sensitivity. Nevertheless, use of an array-based approach is likely to become the most efficient method to identify allele-specific splicing effects at a whole genome level.

For the splicing phenotypes, our experiments using the minigene system suggest that the SNP closest to the intron–exon boundary that shows correlation with the splicing phenotype is very likely to be the functional element. For four of the genes in this study there were additional SNPs in complete LD with the SNP nearest the intron–exon boundary, and although most were over 2 kb away from the exon-skipping event it is possible that the presence of these SNPs influence the splicing process. Further work is needed to define the consequences of the loss of these exons on the functional activities of the encoded protein isoforms and in the levels of expression. There is already evidence that biological consequences of the alternative splicing event we describe in *CD46* are likely to be important. CD46 is a cell-surface glycoprotein involved in regulation of complement activation and it acts as a receptor for several pathogens including measles virus, *Streptococcus pyogenes, Neisseria gonorrhea,* and Neisseria meningitidis [25]. CD46 is known to have two protein isoforms with distinct cytoplasmic tails of 16 or 23 amino acids generated by alternative splicing of exon 8 [26]. These different tails have pivotal effects on the intracellular precursor processing of, and signal transduction by, the CD46 protein [26–28]. We have demonstrated that the inclusion of exon 8 is strongly associated with the presence of a nearby SNP (rs2724374), and whether or not this SNP is directly functionally responsible for the pattern, rs2724374 is a genetic marker for what appears to be an important functional protein isoform. Variants of *CD46* have been associated with outcome in hemolytic uremic syndrome [29], but genetic association studies using the rs2724374 SNP have not been reported. For *CASP3* we have shown that, in individuals who are heterozygous for the splicing SNP rs4647603, there appears to be compensatory upregulation of expression of the full-length *CASP3* isoform derived from the other chromosome. This suggests some functionally important difference between the two *CASP3* isoforms. The consequences of the allele-specific splice events we have defined are summarised in Table 2.

**Table 2:**
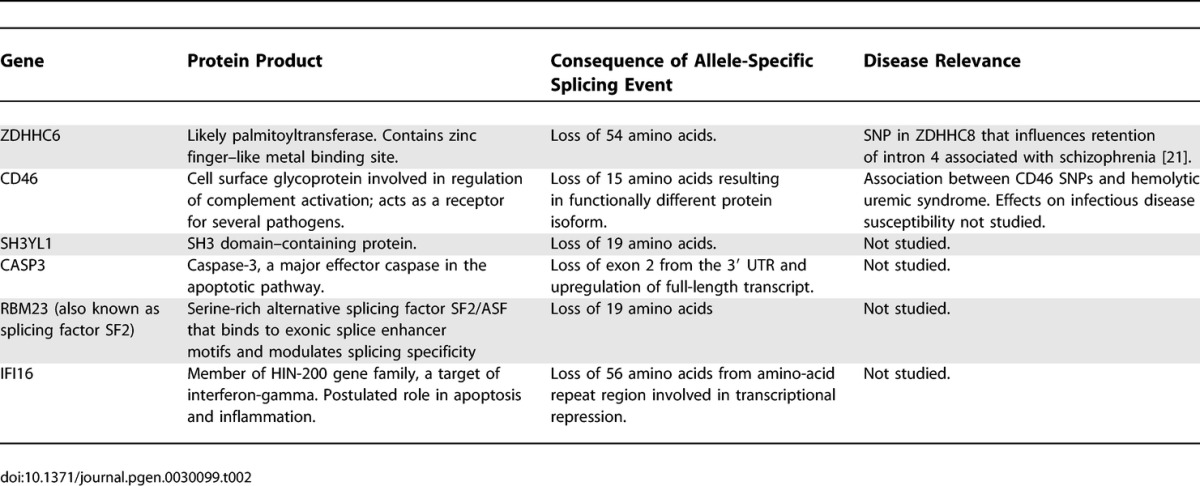
Biological Consequences of Identified Allele-Specific Alternative Splicing Events

In this study we focused on only one form of splicing variation in a relatively small number of genes. Larger-scale whole genome studies investigating additional splicing patterns, such as alternative donor and acceptor sites, will be needed to determine the extent of SNP-associated splicing phenotypes. Our findings raise the possibility that SNP effects on splicing may be at least as prevalent in the genome as those on overall gene expression [11]. SNPs that predict splicing phenotypes are likely to be important markers to include in genetic association studies of complex diseases.

## Materials and Methods

### Exon selection.

A number of different publicly available databases of observed mRNA transcripts are available. We used the EBI-ASD (www.ebi.ac.uk/asd), which is a database of computationally delineated alternative splice events derived from alignments of expressed sequence tags or cDNA sequences with the corresponding genomic sequences for each gene. Using this resource, we identified transcripts where at least two isoforms are detected in which complete exons (called simple cassette exon events on the EBI-ASD) are skipped. These events generally result in transcripts that differ sufficiently in size to be readily distinguished by simple agarose electrophoresis. Primers were designed in the flanking exons, and product sizes for the full length and exon-skipped products were calculated.

### Cell lines.

LCLs from 22 unrelated CEPH individuals selected from the HapMap collection were obtained from the Coriell Institute for Medical Research. Cells were cultured at 37 °C in a 5% CO_2_ environment using RPMI 1640 cell culture medium with 10% fetal calf serum, 200 mM L-glutamine, penicillin, and streptomycin. Cell density was maintained between 200,000 and 800,000 cells/ml. DNA and RNA were each extracted from 10 million cell aliquots. Constitutive expression levels in CEPH cell lines were defined for pooled RNA from four LCLs using an Affymetrix human U133A expression microarray (Affymetrix, http://www.affymetrix.com).

### RNA and cDNA synthesis.

RNA was extracted from cell pellets using TRIREAGENT (Sigma-Aldrich, http://www.sigmaaldrich.com), chloroform, isopropanol, and ethanol precipitation. Total RNA was quantified using UV spectrophotometry. mRNA was extracted from 20 μg of total RNA aliquots using the Dynabeads mRNA purification kit (Invitrogen, http://www.invitrogen.com), and was cDNA synthesised using Stratascript reverse transcriptase (Stratagene, http://www.stratagene.com) with oligo(dT) primers. 1 μl of cDNA was derived from 100 ng of total RNA. Parallel reverse transcriptase negative controls were generated in all cDNA syntheses. PCRs were carried out at standard conditions (30 cycles, melting at 94 °C, annealing at 58 °C, and extension at 72 °C, each for 30 s) using BioTaq DNA polymerase (Bioline, http://www.bioline.com). Primers were designed in the exons flanking the simple cassette exon event. Two products of different lengths were predicted to be amplified, one including the cassette exon and a shorter product lacking the cassette exon. The products were resolved on 2% agarose gels.

### Detecting variation among samples.

Pooled cDNA from all 22 cell lines was used to test each set of primer pairs. Identification of the expected full length product and the shorter product lacking the cassette exon (and no other products) was used to confirm that the predicted alternative splicing event was detectable in our experimental system. Primer sets showing the two expected RT-PCR products were subsequently taken forward to determine if there was variation in the proportion of the two products among different individual cell lines. Detection of variation among cell lines was carried by performing RT-PCR on RNA from each cell line separately. For each cell line, the relative amount of each of the two RT-PCR products (representing the full length and skipped mRNA) was quantified using image analysis of the products visualised on ethidium bromide gels (ImageQuant software; Amersham Biosciences, http://www4.gelifesciences.com). Since both RT-PCR products were amplified by the same primer sets, the RT-PCR was truly competitive, allowing the accurate determination of their relative abundance [30]. To assess the robustness of the ethidium bromide–based quantification method it was compared with quantification using a fluorescence-based technique. Primer pairs with a 5′ FAM modification were used to amplify exon-skipping events from six different genes using cDNA from nine different LCLs. The amplicons ranged in size from 137 bp to 617 bp. The relative amounts of PCR products for each of the 54 reactions were quantified using GeneScan software (Amersham Biosciences). The ratios of quantified products from this method showed excellent correlation with those derived from image quantification of ethidium bromide–stained gels (correlation coefficient 0.93).

We determined the sensitivity of the ethidium bromide quantification system using known starting concentrations of DNA fragments of different lengths, and then quantifying the resulting amplicons. We were able to show that over a range of different product signal intensities, differences in the ratios of the different sized starting material of 10% or greater could be detected reliably (Figure S3). The sensitivity of this method was independent of cycle number.

Each of the 22 cell line samples was assayed in duplicate. The mean ratio of the abundance of the two RT-PCR products from each primer set was calculated for each cell line. When the relative abundance for an individual cell line differed by more than 10% from the average value for the full set of 22 samples, the experiment was repeated using a fresh aliquot of cell culture material. Those events that gave consistent differences in the repeat analysis were then analysed further.

### SNP identification.

Genotypes for SNPs positioned within 250 kb on either side of the exon-skipping event were downloaded from the International HapMap Project Web site (http://www.hapmap.org) [3]. For each event with reproducible variation, we also resequenced the skipped exon and 150 base pairs of the flanking introns to determine if additional SNPs close to the event could be identified, using DNA from each of the 22 LCLs. Sequencing was carried out using purified PCR products generated with M13-tagged primers.

### Genotype correlation analysis.

For each splicing event with reproducible variation, we calculated Pearson's correlation between the ratio of band intensities for the two RT-PCR products and the SNP genotype. In this analysis, we have assumed that any functional SNPs will be *cis*-acting, and thus expect to see an effect that is more pronounced in homozygotes than in heterozygotes. Thus, for the purposes of the correlation analysis, genotypes were coded 1, 2, and 3 to represent the genotypes AA, Ab, and bb, where A represents the major allele and b represents the minor allele. The value of Pearson's correlation was determined for each SNP in each 500-kb region.

### Splice site analysis.

Splice donor and acceptor sequences were scored using a position specific score matrix (PSSM) method [31]. Alignments of mRNA and EST sequences to the reference human genomic assembly (version: hg17) were taken from the University of California Santa Cruz genome database (http://genome.ucsc.edu) and used to define a population of well-supported (appearing in more than nine transcripts) constitutive splice sites. These were used to train the PSSMs, considering three exonic, six intronic nucleotides at the splice donor, and three exonic, 18 intronic nucleotides for the splice acceptor PSSM [31]. We compared the splice site scores of the 250 exons predicted by EBI-ASD to show exon skipping (subdivided into those that showed exon skipping in our experimental model and those that did not) with the splice site scores of 7,431 exons that were always found in mRNA transcripts (constitutively present) randomly selected from the genome. We sought to determine if splice site strength could predict those exons that were likely to be skipped. We also sought to determine if the SNP density near to the intron–exon boundaries differed in those exons that showed alternative splicing compared to those that did not. Finally, the sequence context of SNPs correlated with specific splice patterns was analysed to determine whether they affected know splice enhancer or silencer elements, using four published algorithms: http://ast.bioinfo.tau.ac.il/ESR.htm [32], http://genes.mit.edu/burgelab/rescue-ese [33], http://cubweb.biology.columbia.edu/pesx [34], and http://rulai.cshl.edu/tools/ESE [35].

### Minigene analysis.

Both allelic forms of the SNPs showing correlation with splice patterns in the *ZDHHC6* and *SH3YL1* genes were cloned into a minigene splicing vector (pALTER MAX modified splice vector, http://www.promega.com). Within this modified splicing vector, the multiple cloning site (MCS) of the conventional pALTER MAX minigene vector was replaced by an insert, so that the MCS falls within an intron instead of being within expressed sequence. The new insert contains the 5′ donor splice site from the human β-globin gene intron 1, the MCS, and the 3′ acceptor splice site from the intron of an immunoglobulin gene. When a PCR product with primers designed within introns is used, all donor and acceptor splice sites are present and thus the construct is spliced correctly. The fragments cloned consisted of the exon plus an average of 180 bases of flanking intron. All inserts were confirmed by fluorescent sequencing. HEK293T cells were transfected following the manufacturer's protocol (FuGENETM6, Boehringer Mannheim). Cells (3 × 10^5^) were transfected with 1 μg of DNA and were harvested after 48 h. The relative abundance of the full length and alternatively spliced mRNA derived from the plasmid was analysed using the same methodology as described for the CEPH cell RNA.

### Allele-specific transcript quantification.

Allele-specific differences in *CASP3* expression were determined using a transcribed marker polymorphism (rs4647603) in the exon of interest to distinguish the relative abundance of transcript containing this exon arising from the two alleles. Sixteen unrelated CEPH individuals heterozygous for the transcribed marker were selected from the HapMap collection and obtained from the Coriell repository. RNA and cDNA from each individual were prepared as described above. DNA was extracted using a purification kit (Blood and Cell Culture DNA, Nucleon BACC2; Tepnel, http://www.tepnel.com). For each individual, data were obtained from nine replicates from each of two independent cultures. Allele-specific transcript quantification was carried out by single nucleotide primer extension and MALTI-TOF analysis using a SpectroREADER MassArray (Sequenom, http://www.sequenom.com) mass spectrometer as described previously [7]. RNA ratio values were normalized with the ratios observed for genomic DNA.

## Supporting Information

Figure S1 Relative Transcript Abundance Grouped by SNP Genotype for Nine Additional Unrelated Cell Lines The ratio of transcript abundance (skipped product/full length product) for each of the six alternative splicing events was accurately predicted by the SNP genotype.
(355 KB DPF)

Figure S2 Allele-Specific Differences in *CASP3* Expression Allelic imbalances were determined using an exonic polymorphism to distinguish the relative abundance of transcript arising from the two alleles (G/A) in 16 unrelated CEPH heterozygous individuals. RNA ratios were normalized with the DNA ratios and the data plots represent the average from two independent experiments. Variability between biological replicas was small (mean of relative difference of 9%)
(237 KB DPF)

Figure S3 Sensitivity of Detection Assay Relationship between the measured ratios of band intensity of 2 fragments of DNA after amplification using competitive PCR compared with ratios of the two fragments in the starting material. The two DNA templates were themselves PCR products of different sizes (250 and 463 bp) amplified with M13-tagged primers. These PCR products were diluted and quantified using the picogreen system. A range of different ratios of each of the starting templates was then generated by mixing different volumes together. The mixed samples were then amplified in a single reaction using the M13 primer set, generating two products of different lengths. The products were run out on agarose gels stained with ethidium bromide and visualised with ultraviolet light. Digital photographs of the images were quantified using ImageQuant software (Amersham Biosciences). Each point on the graph represents the mean of eight measurements for each ratio; the bars show 95% confidence intervals. The assay is designed to be sensitive to changes in relative abundance rather than to detect actual molar ratios. Thus, for example, an assay result showing a measured ratio of 3:1 compared with a known ratio of 1:1 does not affect the sensitivity of the assay to detect differences in actual starting concentrations.
(257 KB DPF)

### Accession Numbers

The National Center for Biotechnology Information (NCBI) Entrez Gene (http://www.ncbi.nlm.nih.gov/entrez/query.fcgi?db=gene) accession numbers for the genes discussed in this paper are *CASP3,* 836; *CD46,* 4179; *IFI16,* 3428; *RBM23,* 55147; *SH3YL1,* 26751; and *ZDHHC6,* 64429.

## Abbreviations

CEPH: Centre d'Etude du Polymorphisme Humain
EBI-ASD: European Bioinformatics Institute Alternative Splicing Database
LCL: lymphoblastoid cell line
LD: linkage disequilibrium
RT-PCR: reverse transcriptase PCR
SNP: single nucleotide polymorphism
