# Supplementary material for: Identification of Common Genetic Variation That Modulates Alternative Splicing
Source: PLoS Genet. 2007 Jun 15;3(6):e99. doi: 10.1371/journal.pgen.0030099 (PMC1904363; doi:10.1371/journal.pgen.0030099)
Supplement: Supplementary file 1 [file pgen.0030099.sg001.pdf]

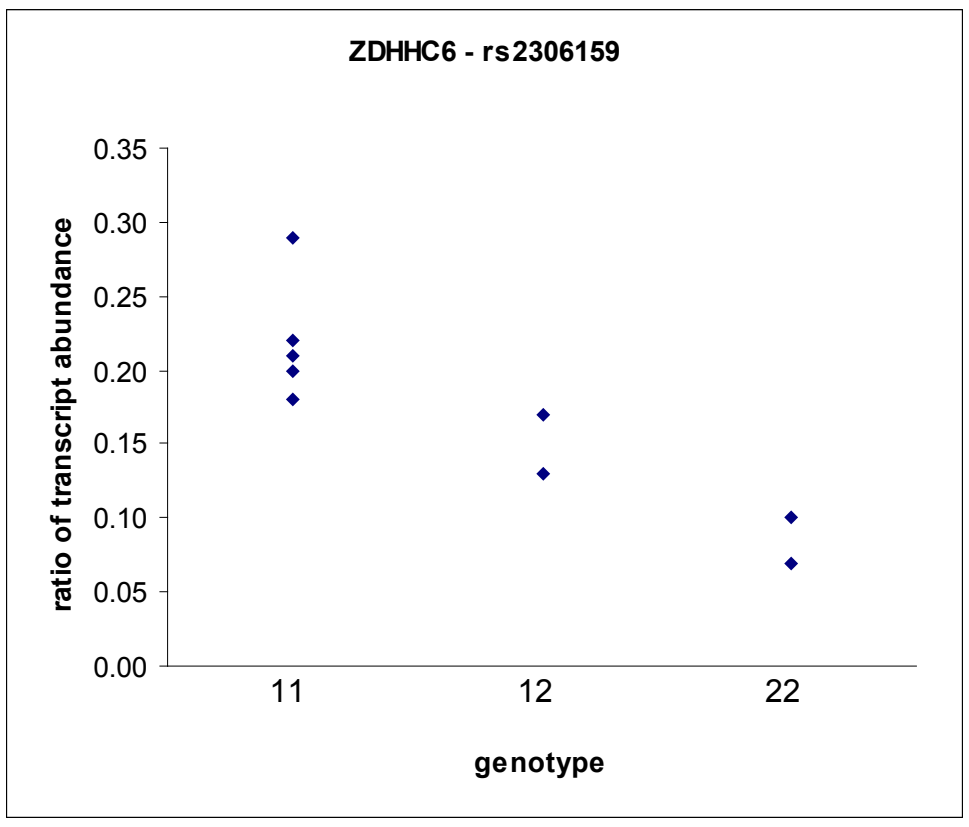
ZDHHC6 - rs2306159
ratio of transcript abundance
0.00
0.05
0.10
0.15
0.20
0.25
0.30
0.35
11
12
22
genotype

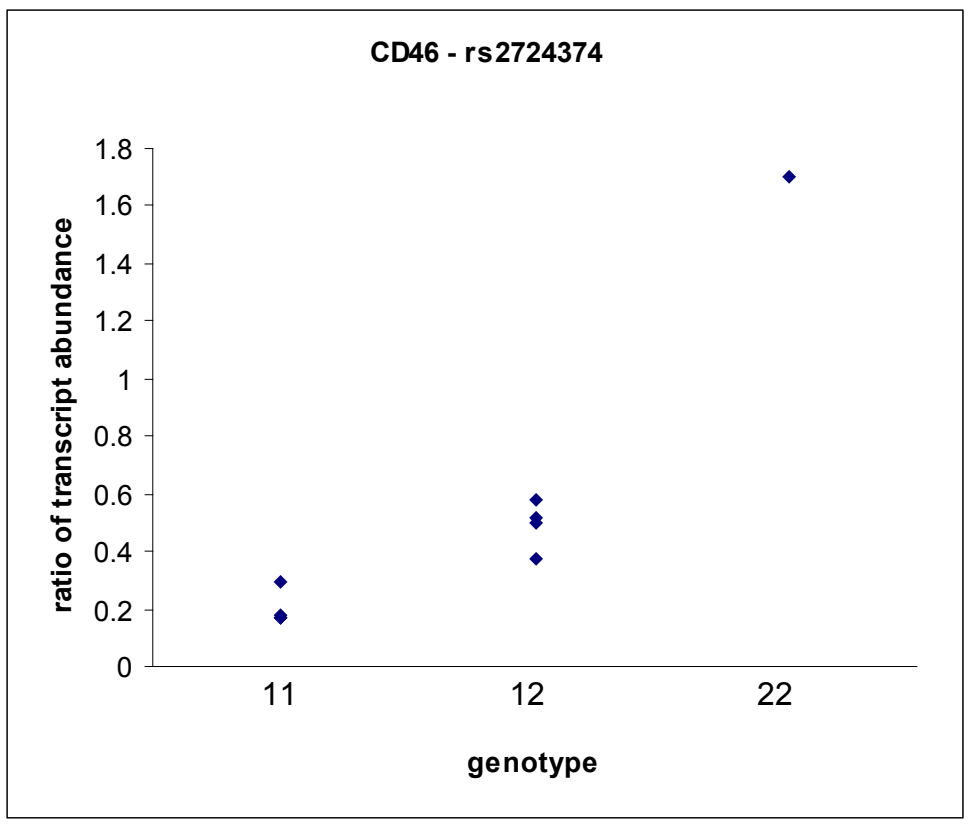
CD46 - rs2724374
ratio of transcript abundance
0
0.2
0.4
0.6
0.8
1
1.2
1.4
1.6
1.8
11
12
22
genotype

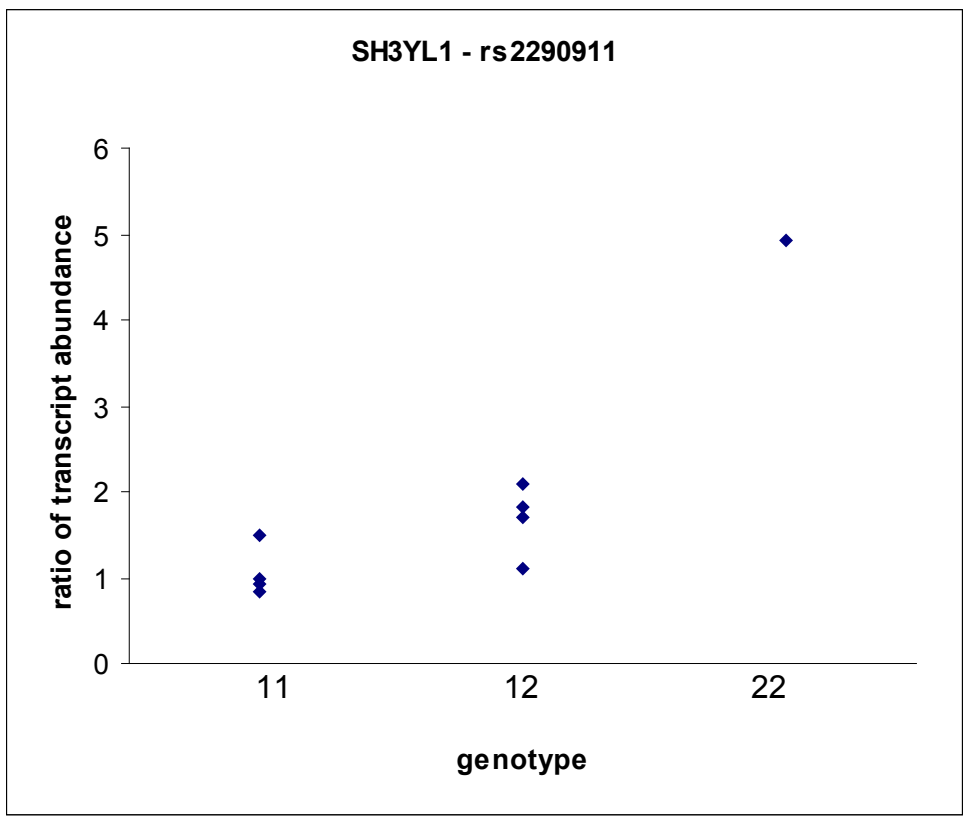
SH3YL1 - rs2290911
ratio of transcript abundance
0
1
2
3
4
5
6
11
12
22
genotype

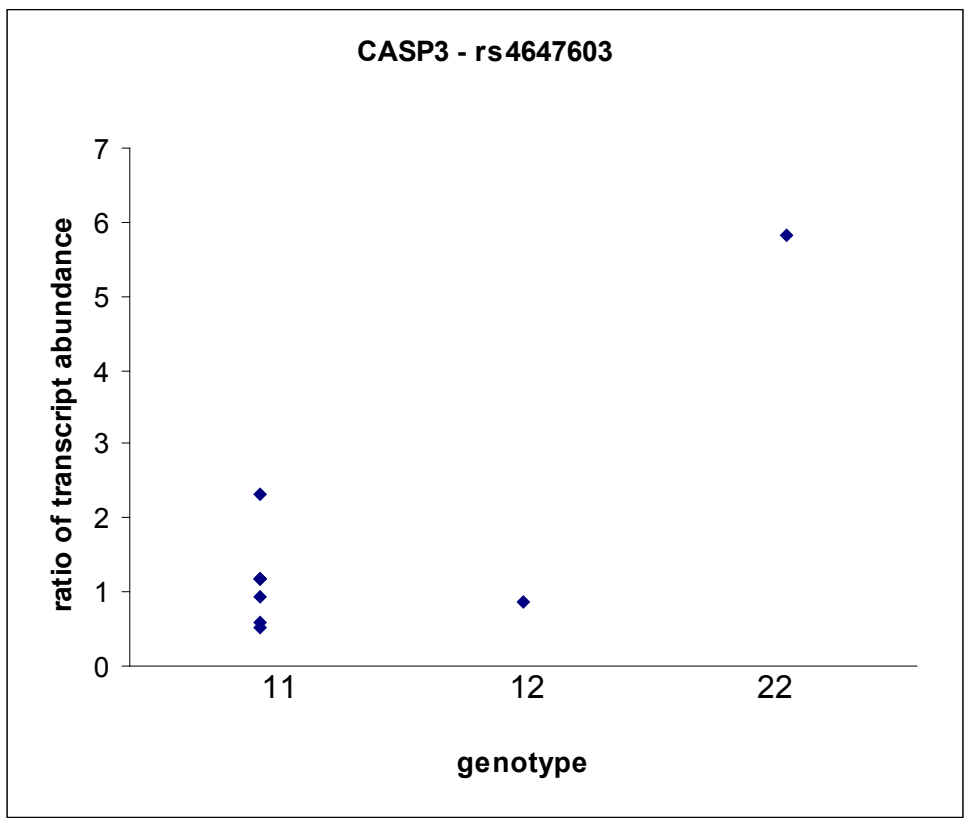
CASP3 - rs4647603
ratio of transcript abundance
0
1
2
3
4
5
6
7
11
12
22
genotype

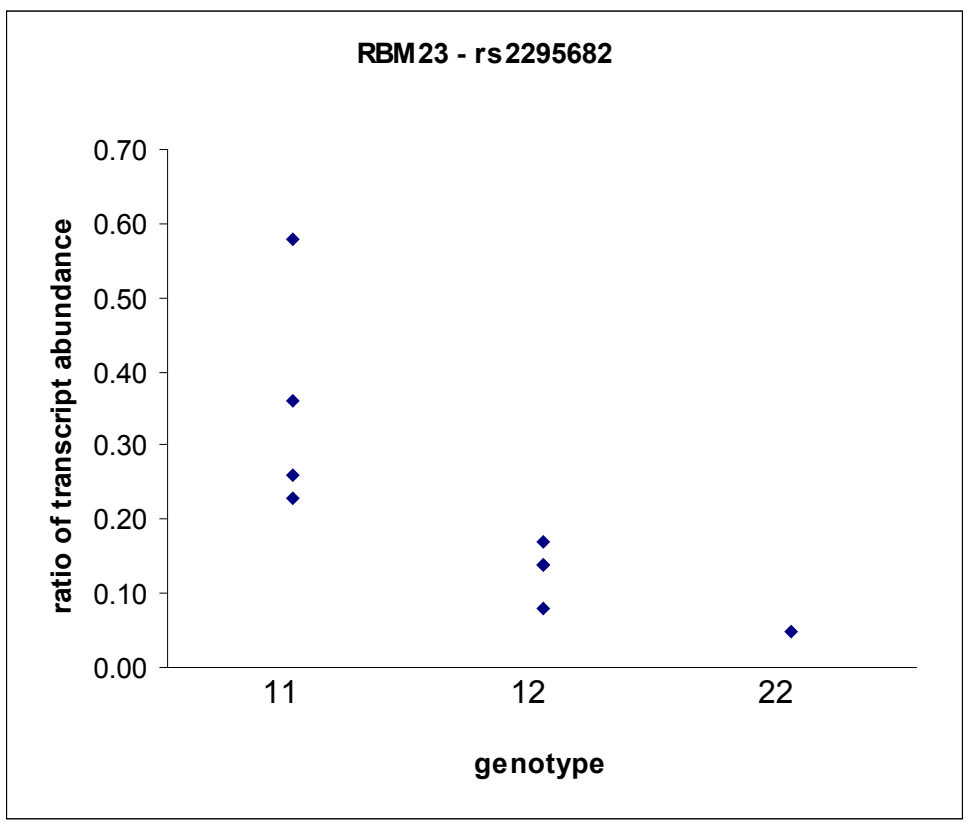
RBM23 - rs2295682
ratio of transcript abundance
0.00
0.10
0.20
0.30
0.40
0.50
0.60
0.70
11
12
22
genotype

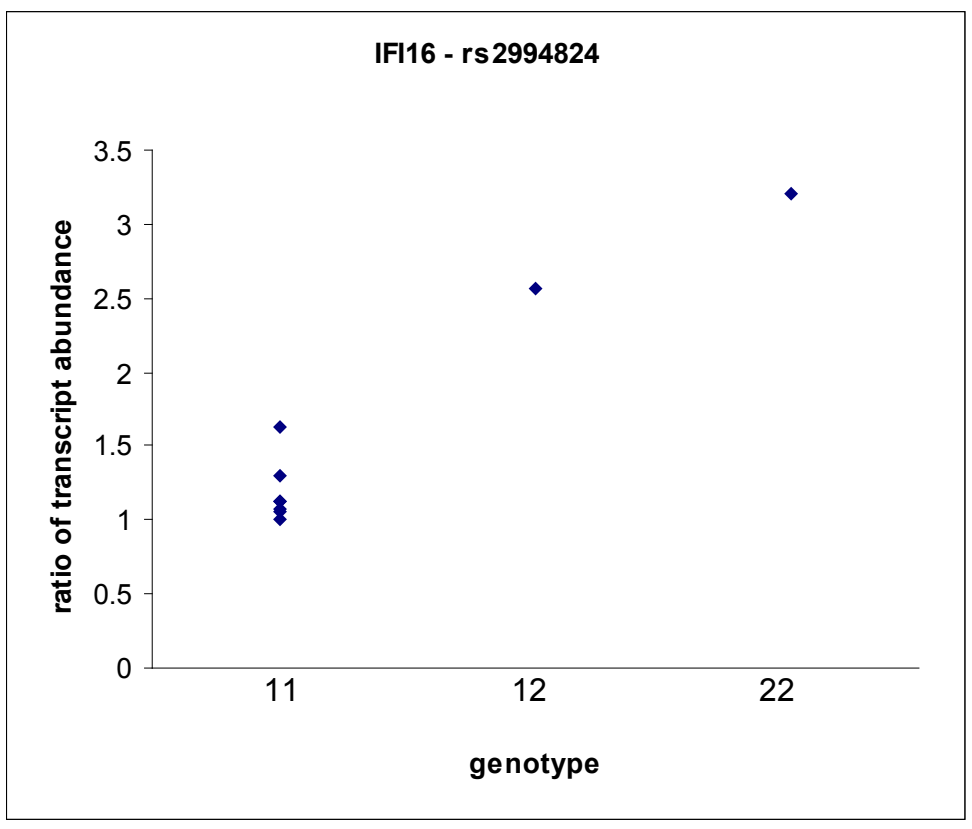
IFI16 - rs2994824
ratio of transcript abundance
0
0.5
1
1.5
2
2.5
3
3.5
11
12
22
genotype
