# Supplementary material for: Identification of Common Genetic Variation That Modulates Alternative Splicing
Source: PLoS Genet. 2007 Jun 15;3(6):e99. doi: 10.1371/journal.pgen.0030099 (PMC1904363; doi:10.1371/journal.pgen.0030099)
Supplement: Supplementary file 2 [file pgen.0030099.sg002.pdf]

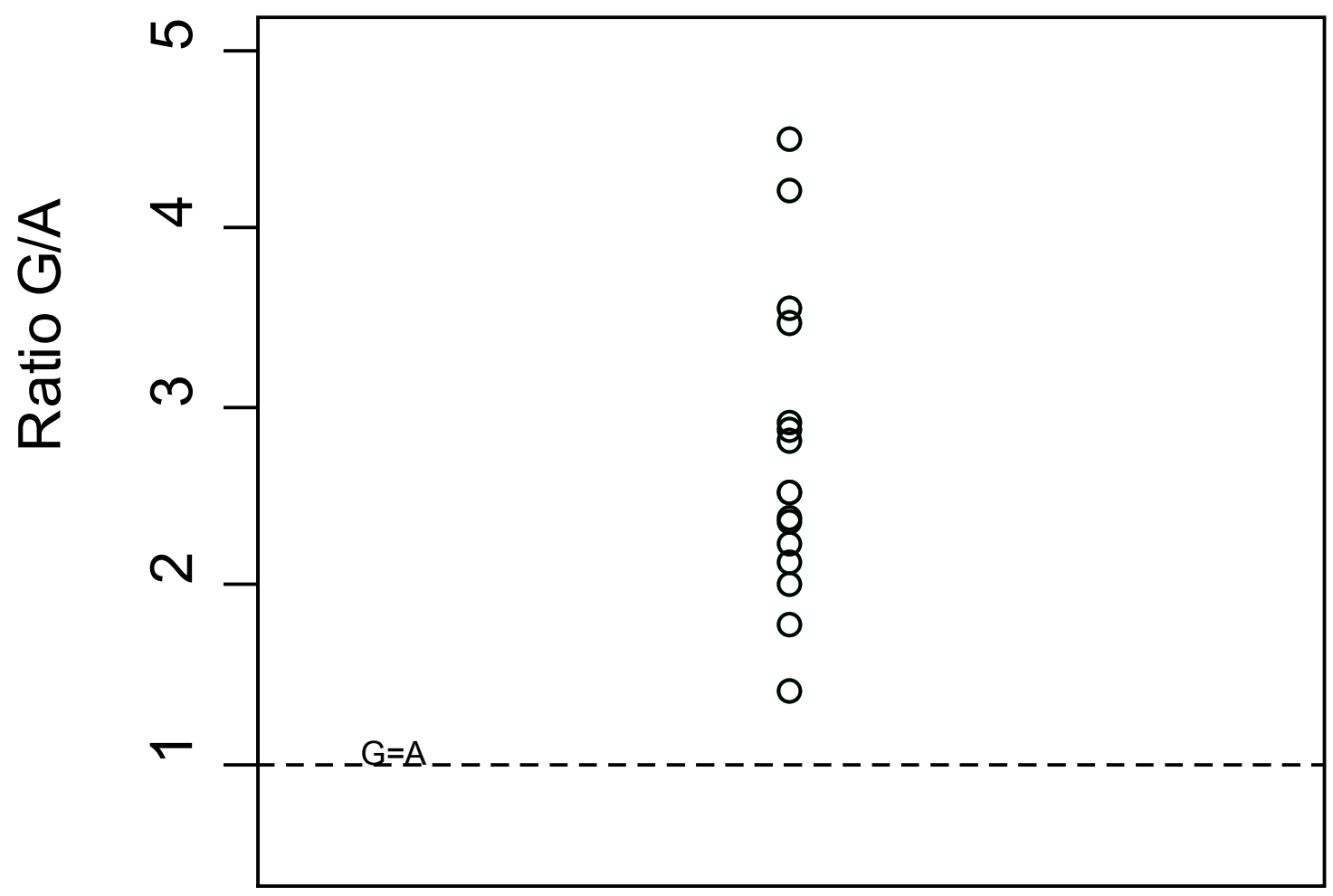
Ratio G/A
5
4
3
2
1
G=A
