# Supplementary material for: Identification of Common Genetic Variation That Modulates Alternative Splicing
Source: PLoS Genet. 2007 Jun 15;3(6):e99. doi: 10.1371/journal.pgen.0030099 (PMC1904363; doi:10.1371/journal.pgen.0030099)
Supplement: Supplementary file 3 [file pgen.0030099.sg003.pdf]

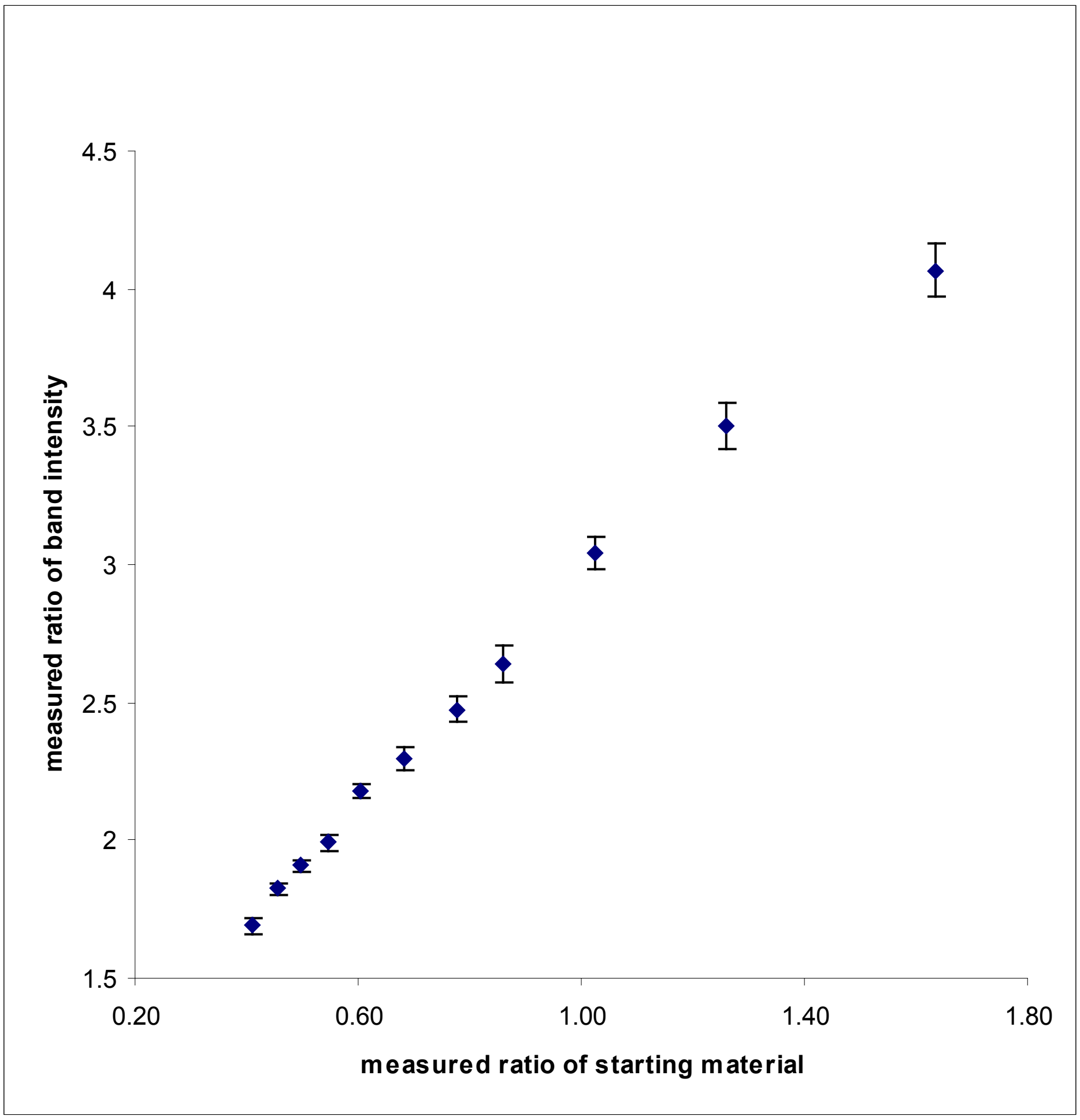
4.5
4
3.5
3
2.5
2
1.5
0.20
0.60
1.00
1.40
1.80
measured ratio of band intensity
measured ratio of starting material
